# Genome-Wide Association Analysis Unravels New Quantitative Trait Loci (QTLs) for Eight Lodging Resistance Constituent Traits in Rice (*Oryza sativa* L.)

**DOI:** 10.3390/genes15010105

**Published:** 2024-01-16

**Authors:** Ognigamal Sowadan, Shanbin Xu, Yulong Li, Everlyne Mmbone Muleke, Hélder Manuel Sitoe, Xiaojing Dang, Jianhua Jiang, Hui Dong, Delin Hong

**Affiliations:** 1 State Key Laboratory of Crop Genetics & Germplasm Enhancement and Utilization, Nanjing Agricultural University, Nanjing 210095, China; ognigamalsowadan@yahoo.fr (O.S.); 2020201002@stu.njau.edu.cn (S.X.); 2018101068@njau.edu.cn (Y.L.); mmboneve@gmail.com (E.M.M.); heldermsitoe@gmail.com (H.M.S.); donghui@njau.edu.cn (H.D.); 2 Institute of Crop Research, Anhui Academy of Agricultural Sciences, Hefei 230031, China; 3 Department of Agriculture and Land Use Management, School of Agriculture, Veterinary Sciences and Technology, Masinde Muliro University of Science and Technology, Kakamega P.O. Box 190-50100, Kenya; 4 Faculty of Agronomy and Biological Sciences, Púngue University, P.O. Box 323, Manica 2202, Mozambique; 5 Institute of Rice Research, Anhui Academy of Agricultural Sciences, Hefei 230031, China; danxj@aaas.org.cn (X.D.); jiangh@aaas.org.cn (J.J.)

**Keywords:** *Oryza sativa*, lodging resistance, stem anti-thrust, QTLs, internode length, favorable allele

## Abstract

Lodging poses a significant challenge to rice yield, prompting the need to identify elite alleles for lodging resistance traits to improve cultivated rice varieties. In this study, a natural population of 518 rice accessions was examined to identify elite alleles associated with plant height (PH), stem diameter (SD), stem anti-thrust (AT/S), and various internode lengths (first (FirINL), second (SecINL), third (ThirINL), fourth (ForINL), and fifth (FifINL) internode lengths). A total of 262 SSR markers linked to these traits were uncovered through association mapping in two environmental conditions. Phenotypic evaluations revealed striking differences among cultivars, and genetic diversity assessments showed polymorphisms across the accessions. Favorable alleles were identified for PH, SD, AT/S, and one to five internode lengths, with specific alleles displaying considerable effects. Noteworthy alleles include RM6811-160 bp on chromosome 6 (which reduces PH) and RM161-145 bp on chromosome 5 (which increases SD). The study identified a total of 42 novel QTLs. Specifically, seven QTLs were identified for PH, four for SD, five for AT/S, five for FirINL, six for SecINL, five for ThirINL, six for ForINL, and four for FifINL. QTLs *qAT/S-2*, *qPH2.1*, *qForINL2.1*, and *qFifINL* exhibited the most significant phenotypic variance (PVE) of 3.99% for the stem lodging trait. AT/S, PH, ForINL, and FifINL had additive effects of 5.31 kPa, 5.42 cm, 4.27 cm, and 4.27 cm, respectively, offering insights into eight distinct cross-combinations for enhancing each trait. This research suggests the potential for crossbreeding superior parents based on stacked alleles, promising improved rice cultivars with enhanced lodging resistance to meet market demands.

## 1. Introduction

Rice (*Oryza sativa* L.) is a significant cereal for global food security [1]. Nonetheless, rice production faces a substantial challenge from lodging. The stem lodging of rice plants is the bending or breaking of stems due to environmental stresses or insufficient strength to support the weight of the panicles during the grain-filling stage. The lodging of rice plants has detrimental effects on the structure and function of the crop canopy, which hampers the movement of nutrients and water, thereby assimilating through the vascular bundles and reducing assimilation during grain filling [2]. The development of mechanization and standardization in rice production makes rice varieties susceptible to lodging, which is a concern as it hinders mechanized harvesting.

Stem lodging traits include plant height, stem diameter, anti-thrust, and internode length, which determines the overall stem height. Taller rice plants are more prone to lodging, a phenomenon where the plants bend or break under environmental factors such as wind or heavy rain [3,4]. A greater stem diameter provides structural support and strength to the plant, reducing the risk of bending or breaking. A thicker stem can better withstand external pressures, contributing to overall plant stability [5]. On the other hand, increased pushing resistance indicates a stronger and more rigid stem at the lower part of the plant, offering better resistance against external forces and minimizing the likelihood of lodging [6]. The length of internodes is another factor that affects the overall plant architecture and stability. This prevalent issue detrimentally impacts the harvest quantity and quality [7]. Elevated pushing resistance at the second internode length (2 inl) contributes to a decreased lodging risk in rice. Higher pushing resistance at the third (3 inl), fourth (4 inl), and fifth (5 inl) internode lengths is associated with reduced lodging in rice [8,9].

Through genetic mapping and genome-wide association studies (GWAS), several quantitative trait loci (QTLs) associated with lodging traits, including plant height, stem diameter, and stem anti-thrust, have been identified across rice populations and cultivars [10,11]. Researchers have identified numerous quantitative trait loci (QTLs) responsible for regulating plant height in rice, with some of these QTLs having known functions and specific genetic locations. These QTLs are distributed across all 12 chromosomes, with 342 QTLs detected. Notably, 64, 55, 27, 27, 21, 23, 26, 37, 24, 11, 13, and 14 QTLs are located on chromosomes 1 to 12, respectively [12,13,14,15]. Many of these QTLs significantly affect plant height and have been consistently found in different populations. One such QTL, qPH1, has been of particular interest and is associated with a putative cytochrome P450 encoded by the D2 gene responsible for the rice dwarf mutant [12]. Additionally, two functional genes, OsMAPK6 and D35, have been identified and are colocalized with QTLs qPH6-2 and qPH6-4 [13]. Additionally, the confidence intervals of qPH1.2 and qPH8 contain known plant height genes SD1 and OsSPY, respectively [14]. The EUI1 gene, discovered on chromosome 5, encodes a unique P450 monooxygenase that regulates the levels of bioactive gibberellin, ultimately affecting plant height. Overexpression of EUI1 leads to a significant reduction in plant height [15].

One of the key factors contributing to lodging is the length of internodes, which affects the overall plant architecture and stability [7]. The SBI (shortened basal internodes) gene on chromosome 5 encodes OsGA2ox and primarily influences basal internodes, resulting in reduced plant height [16]. Additionally, on chromosome 1, the *Ssi1* gene, known as short second internode 1, controls the elongation of the second internode and plays a role in semi-dwarfism and high lodging resistance [17]. Wang et al. [18] detected 13 QTLs related to the fourth internode length, while Wang et al. [19] identified two significant QTLs, qFOIL-6-4 and qFOIL-7-5, contributing to the phenotypic variance.

In the context of stem diameter in rice, multiple quantitative trait loci (QTLs) have been identified on the 12 chromosomes, specifically on chromosomes 1 and 6. These QTLs are associated with the diameters of various internodes and are genetically linked to the semidwarf1 (*sd1*) gene. Notably, a major-effect QTL named *q2ID1* was found to overlap with the *sd1* gene [20]. Another *gw2* mutation has been found to enhance grain productivity and lodging resistance in rice by simultaneously promoting thicker internodes.

In studying pushing resistance in rice, a comprehensive examination of quantitative trait loci was undertaken within a population of backcross inbred lines derived from the *japonica* Nipponbare × *indica* Kasalath cross. Notably, among the five QTLs identified as associated with pushing resistance in rice, it was noteworthy that only one, denoted as prl5, located on chromosome 5, exhibited a positive effect [21].

In this comprehensive study, we aim to present findings on elite alleles associated with the five internode lengths (first internode (FirINL), second internode (SecINL), third internode (ThirINL), fourth internode (ForINL), and fifth (FifINL)), plant height (PH), and stem diameter (SD), as well as stem anti-thrust (AT/S), using a natural population of rice consisting of 518 accessions and their potential implications in enhancing lodging resistance. QTLs associated with internode elongation are herein reported, as well as the subsequent design of parental combination for cultivar improvement.

## 2. Materials and Methods

### 2.1. Sample Collection and Field Experiments

In this study, a comprehensive collection of 518 rice accessions was utilized. Among these, 400 accessions were obtained from China, 102 from Vietnam, and 16 from Japan (Supplementary Table S1). These varieties, originating from different regions of the respective countries, have been extensively employed as parental lines in plant breeding endeavors over the past few decades. The seeds for all accessions were procured, stored, and provided by the State Key Laboratory of Crop Genetics and Germplasm Enhancement and Utilization at Nanjing Agricultural University (Nanjing, China). The 518 rice accessions were planted in a field located at the Institute of Rice Research, Anhui Academy of Agricultural Sciences, Hefei, China (117°17′ E, 31°52′ N). The field experiments were conducted over two years. The process involved sowing dry seeds in seedling beds during the first week of May 2021 and 2022. At a growth stage of 30 days, the seedlings were transplanted into the paddy field in June of each year. The experiment employed a randomized complete block layout with two replications. The transplanting was performed in rows, with each row comprising eight plants at a spacing of 20 cm × 20 cm. Standard agronomic practices were consistently applied to all the plots throughout the study.

### 2.2. Phenotypic Investigation for Eight Lodging Resistance Traits

At the maturity stage, three plants in the middle rows of each plot from each replication were randomly sampled for measurements of the eight traits.

#### 2.2.1. Stem Anti-Thrust

Single-stem anti-thrust was used as the evaluation index of lodging resistance. A plant lodging tester (Supplementary Figure S1a) was pressed 20 cm from the base of the plant. Pressure was exerted perpendicularly to the rice plant stem to bend the rice stem to a 45° tilt angle (measured by a protractor) (Supplementary Figure S1b). The plant lodging tester automatically recorded the maximum pressure value, which is the point at which the rice plant achieves maximum pressure resistance. Measurements were taken on a single stem per plant for five plants and then averaged.

#### 2.2.2. Plant Height

This was measured using a ruler from the soil surface to the top of the main spike length. The measurement was recorded in cm.

#### 2.2.3. Stem Diameter and Internode Length

Vernier caliper was employed to measure the stem diameter on the main stem of the second elongated internode, which was recorded in mm (Supplementary Figure S1b).

The five internode lengths under the panicle were considered in this study. The internodes were numbered from 1 to 5, starting from the one immediately after the panicle. The length of each internode was measured using a ruler (cm) (Supplementary Figure S1d).

#### 2.2.4. SSR Marker Genotyping

In this study, researchers utilized existing data on rice molecular mapping and microsatellite data from various sources [22,23,24,25,26] to conduct genotyping using 262 SSR markers (Supplementary Table S2). These markers were distributed across the 12 chromosomes of rice, as shown in Supplementary Figure S2.

To extract genomic DNA for genotyping, the leaf tissue of a plant selected from each of the 518 accessions was used, following a previously described method [27]. The DNA amplification was then performed, with primers synthesized by Shanghai General Biotech Co. Ltd. in Shanghai, China.

For each PCR reaction, a 10 μL mixture was prepared. This mixture contained 20 ng of genomic DNA, 0.14 pM of each forward and reverse primer, 0.1% Triton X-100, 10 mM tris HCl (pH 9.0), 0.5 nM dNTPs, 0.5 U of Taq polymerase, 1.5 mM MgCl_2_, and 50 mM KCl. The amplification PCR was performed using a 2720 Thermal Cycler (AB 2720TC^TM^) MJ Research™ Incorporated, Hampton, NH, USA. The PCR had the following cycling conditions: initial denaturation at 94 °C for 5 min, then 34 cycles of denaturation at 94 °C for 0.5 min, annealing at 55–61 °C for 1 min, extension at 72 °C for 1 min, before a final extension step at 72 °C for 10 min. An 8% polyacrylamide gel was run for one hour at 150 V to visualize the PCR products, and the resultant bands were observed through silver staining.

#### 2.2.5. Heritability

The phenotypic data was carried out using analysis of variance (ANOVA) using the SAS statistical package (SAS Institute Inc., Cary, NC, USA) to gain insights into the observed variations. In order to estimate the broad heritability (*H*_2*B*_) of the traits under investigation, the following formula was employed: *H*_2*B*_ = σ_g_^2^/(σ_g_^2^+ σ_e_^2^/*n*. Here, *H*_2*B*_ represents the heritability, σ_e_^2^ denotes the error variance, σ_g_^2^ signifies the genetic variance, and *n* represents the number of replicates used in the study. This calculation unravels the relative contributions of genetic and environmental factors to the detected variability in the phenotypic traits.

#### 2.2.6. Genetic Phylogenetic and Population Structure Analysis

Gene diversity, summary statistics, allele frequency, number of alleles per locus, and the polymorphism information content (PIC) were assessed using Power Marker V3.25, as described in the following citations [28,29,30,31]. To determine the genetic relationships between individuals, pairwise relatedness coefficients (K, kinship matrix) were computed using SPAGeDi55 (Spatial Pattern Analysis of Genetic Diversity).

The population structure of the 518 accessions was analyzed using the STRUCTURE V2.2 software [32]. To establish the optimal number of populations (K), ten independent runs were executed with a burn-in of 50,000 iterations, followed by 100,000 for each K value ranging from 2 to 10. The STRUCTURE software employs a model-based clustering algorithm to identify sub-clusters with significant allele frequencies. Individuals were assigned to K clusters, which were predetermined but allowed for variation in independent algorithm runs. The most probable number of clusters (K) was chosen based on the logarithmized probabilities of the data [Pr(X|K)] and compared to values obtained using the method described by Pritchard et al. [33]. To examine the genetic relationships among the accessions, an unrooted neighbor-joining phylogenetic tree was constructed via the software Power Marker V3.25 and calculated in MEGA 6.06 [34]. Nei’s distance [35] was computed and utilized for the neighbor-joining reconstruction, as implemented in the viewer software.

#### 2.2.7. Linkage Disequilibrium Analysis

The linkage disequilibrium (LD) was assessed using TASSEL 3.01 software [36]. This analysis evaluated each pair of SSR loci across all rice accessions and clusters derived from STRUCTURE analysis. The modified D′ measures for multiple loci were utilized, and the significance of each SSR pair was determined based on 100,000 permutations. Furthermore, the phenotypes among the groups generated by STRUCTURE were compared using the ANOVA procedure implemented in SAS program version 8 (SAS Institute Inc., Cary, NC, USA).

#### 2.2.8. Association Mapping

In order to avoid potential false associations, a mixed linear model (Q + K) implemented in TASSEL 3.0 [37,38,39,40] was employed for our analysis. This model was used to address the effect of population structure and relatedness among 518 rice accessions. The association between marker alleles and lodging resistance traits was investigated through trait analysis by association and linkage, while considering the overall population structure.

The association analysis was based on the *p* and R^2^ values. The *p* value indicates the association between a marker and a trait, while the R^2^ value represents the proportion of variation elucidated by the identified marker. The non-amplified allele (null allele) was utilized to evaluate the phenotypic effects of other alleles, as suggested in the cited studies [41,42]. Calculating the phenotypic effect of a single allele followed the formula: a_i_ = ∑x_ij_/n_i_ − ∑N_k_/n_k_. Here, a_i_ denotes the phenotypic effect of the i allele, x_ij_ represents the phenotypic measurement values of variety j carrying the i allele, n_i_ is the number of materials carrying the i allele, N_k_ signifies the phenotypic value of variety k carrying the null allele, and n_k_ represents the number of materials carrying the null allele.

## 3. Results

### 3.1. Phenotypic Evaluations

**Figure 1:**
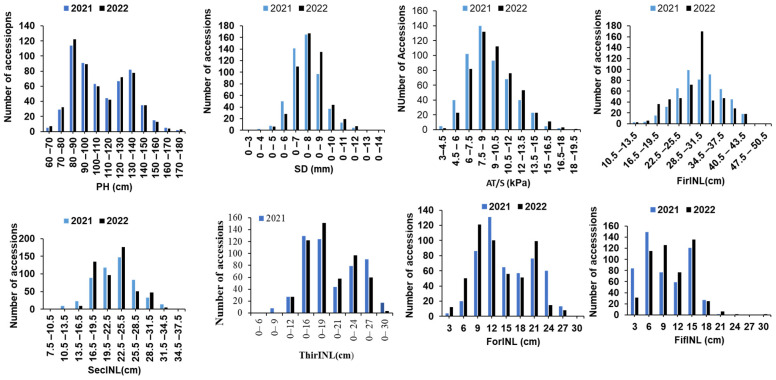
Frequency distribution of plant height and its component traits in the natural population.

The distributions of global averages for the eight traits in each subspecies are depicted in Figure 1. The analysis of variance unveiled highly significant differences among cultivars for each trait, with a statistical significance level of *p* < 0.01. In the 518 accessions, the mean values of AT/S ranged from 9.06 ± 2.47 Kpa to 9.05 ± 2.48 Kpa (Figure 1, Table 1), CV values from 28.34% to 27.44%. Plant height (PH) ranged between 62.22 and 175.5 cm, with mean values of 111 ± 23 cm to 112 ± 23 cm across environments and CV values ranging from 20.62% to 21.79% (Table 1, Figure 1). In stem diameter, accessions exhibited values between 3.69 and 13.1 mm, with mean values ranging from 7.41 ± 1.28 mm to 7.40 ± 1.29 mm and CV values ranging from 17.29% to 15.38% (Table 1, Figure 1).

Internode length FirINL exhibited minimum and maximum ranges of 12.43 and 95.67 cm, respectively, with corresponding mean values ranging from 34.86 ± 7.93 cm to 34.85 ± 7.93 cm and CV values from 22.75% to 20.95% (Table 1, Figure 1). The lowest and highest SecINL ranged from 12.43 and 95.67 cm, with mean values of 23.46 ± 5.24 cm to 23.58 ± 5.22 cm (Table 1, Figure 1) and CV values from 21.35% to 22.16%. ThirINL, on the other hand, recorded lengths of between 10.25 and 39.22 cm, with mean values ranging from 19.31 ± 5.45 cm to 19.48 ± 5.48 cm and CV values from 28.35% to 28.85% (Table 1, Figure 1). With ForINL, values ranged from 1.96 cm to 31.73 cm (Figure 1, Table 1), with CV values from 42.91% to 41.73%. Finally, FifINL exhibited values ranging from 0.94 cm to 29.54 cm, with CV values from 45.35% to 46.63% (Table 1, Figure 1).

### 3.2. Genetic Diversity

Population genetic diversity was assessed using a comprehensive set of 262 SSR markers distributed throughout the rice genome to ensure representative coverage. From this analysis, all 262 SSR markers displayed polymorphism across the diverse set of 518 rice accessions under examination. Subsequently, 2725 alleles were identified across the entire dataset (Supplementary Table S2).

The allele counts varied widely across loci, ranging from 2 (at locus RM437 on chr5) to 25 (RM7545 on chr10), with an average of 10.45 alleles per locus (Supplementary Table S2). The mean genetic diversity exhibited a broad spectrum, ranging from 0.0676 (RM7303 on chr11) to 0.9383 (RM7545 on chr10) (Supplementary Table S2). The Polymorphic Information Content (PIC) averaged 0.7119, with values spanning from 0.0656 (RM7163 on chr11) to 0.9349 (RM7545 on chr10), and the majority of markers fell within the range of 0.5010 to 0.9349 (Supplementary Table S2). Specifically, 230 markers (87.79%) were highly informative (PIC > 0.5), 25 markers (9.54%) displayed moderate informativeness (0.5 > PIC > 0.25), and 7 markers (2.67%) were only slightly informative (PIC < 0.25).

### 3.3. Population Structure and Genetic Relatedness

**Figure 2:**
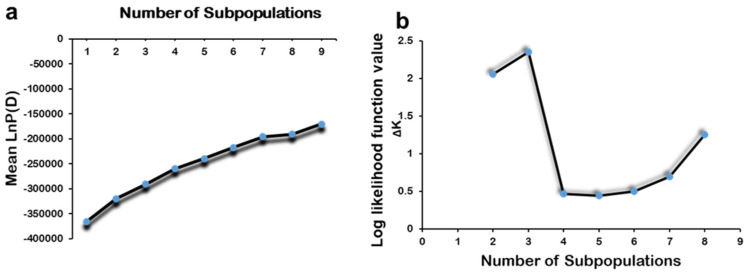
Changes in the log-likelihood function value (**b**) and ΔK values (**a**) with the number of subpopulations.

An analysis employing a model-based approach for population structure assessment (MBPSA) unveiled significant population stratification among the dataset comprising 518 rice accessions. With this analysis, it was observed that the log-likelihood values consistently increased with the augmentation of subpopulation numbers (Figure 2a). Consequently, the diagnostic criterion ΔK proposed by Evanno et al. [43] was employed to ascertain the appropriate number of subpopulations, denoted as “k”. Remarkably, the highest ΔK value was identified at k = 3 compared to other k values. This compelling result led to classifying the 518 accessions into three distinct subpopulations, as depicted in Figure 2b. Utilizing the Q matrix information, each accession with a Q value greater than 0.9 was appropriately assigned to its corresponding subpopulation (detailed in Supplementary Table S1 and Supplementary Figure S2). Of the 518 accessions, 468 were effectively categorized into the three subpopulations, with the remaining 50 assigned to an admixture group. A neighbor-joining tree was constructed according to Nei’s genetic distances (Supplementary Figure S3), which corroborated the findings of the STRUCTURE analysis. Importantly, these clusters exhibited a strong correspondence with the geographical regions of the germplasm, with only minor discrepancies. For example, Sub-Population 1 primarily comprised germplasm from Eastern China, Sub-Population 2 consisted mainly of germplasm from Northeast China, and Sub-Sub-Population 3 primarily included germplasm from Vietnam.

**Figure 3:**
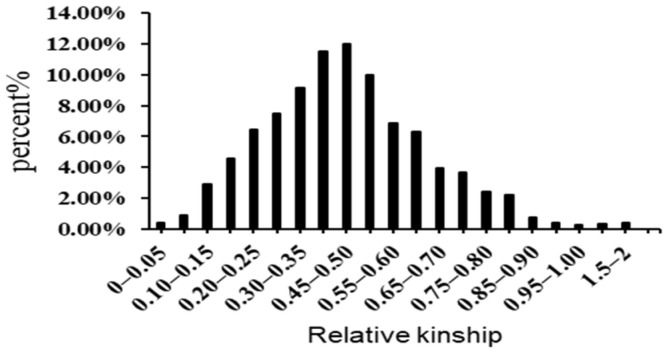
Distribution of pairwise kinship coefficients among 518 rice accessions based on 262 SSR markers.

Furthermore, the genetic relatedness analysis conducted in this study, utilizing 262 genetic SSR markers, revealed that over 89% of the kinship coefficient values (K matrix) fell within the range of 0.35–0.50. The remaining 9.8% of values indicated various degrees of genetic relatedness between pairwise accessions (Figure 3). Additionally, the genetic relatedness analysis results were utilized to derive a K matrix, which subsequently served as a valuable resource for association analysis.

### 3.4. Genetic Differentiation among Subpopulations

**Figure 4:**
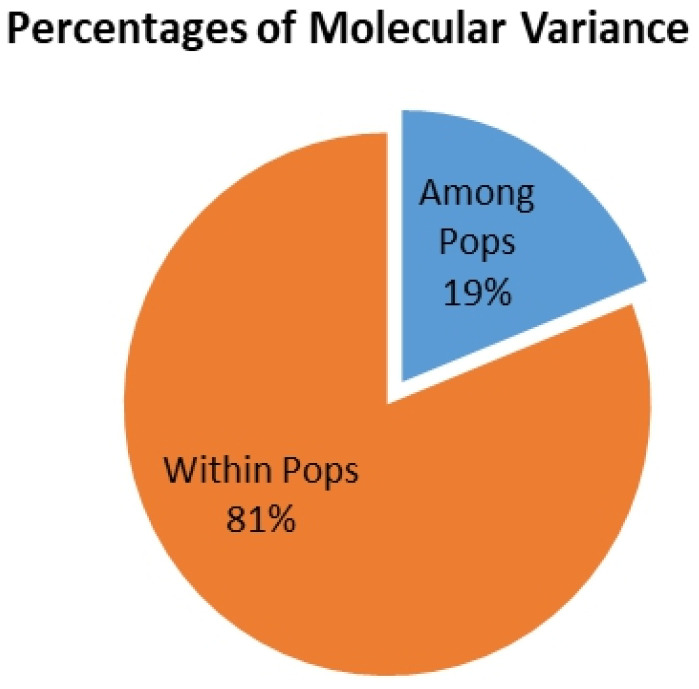
Analysis of molecular variance (AMOVA) for the three subpopulations of rice varieties.

The model-based approach yielded three subpopulations, and an analysis of molecular variance (AMOVA) was performed to evaluate the distribution of genetic variation among and within these subpopulations. The results of the AMOVA revealed that 19% of the total genetic variation was observed among the subpopulations. In comparison, the remaining 81% was attributed to individual differences within each subpopulation (see Table 2 and Figure 4). To further examine the genetic variation within these subpopulations, fixation index (*Fst*) statistics were employed. Pairwise *Fst* values indicated significant differentiation among all pairs of subpopulations, ranging from 0.44 (between Sub-Pop 3 and Sub-Pop 2) to 0.58 (between Sub-Pop 2 and Sub-Pop 3), suggesting that all three subpopulations were significantly distinct from each other (*p* < 0.01). Additionally, the corresponding standard Nei’s genetic distance between the three subpopulations ranged from 0.48 to 0.69, with Sub-Pops 1 and 3 showing more significant differentiation based on the initial estimates (Table 3). Notably, the AMOVA and *Fst* analyses were consistent with the results obtained from phylogenetic tree-based and STRUCTURE analyses, collectively highlighting the presence of statistically moderate genetic diversity and a highly diverse population structure in this study.

### 3.5. Linkage Disequilibrium

The linkage disequilibrium (LD) analysis within the three subpopulations revealed varying levels of significant LD pairwise loci. Among these subpopulations, POP3 exhibited the lowest percentage of significant LD pairwise loci at 2.4%, whereas POP2 displayed the highest percentage at 4.7% (Table 4). The mean D′ value, a measure of LD, was observed to be highest in POP1, standing at 0.64, while the lowest value of 0.54 was recorded in POP3. Regression analysis was performed to delve further into the relationship between D′ values and genetic intra-chromosome distance (syntenic marker pairs). The results of this analysis demonstrated that the genomes of all three subpopulations conformed to the equation y = bln(x) + c (Supplementary Figure S4). Furthermore, the minimal LD decay distance for POP1, POP2, and POP3 was measured at 54.22 cM, 49.96 cM, and 49.20 cM, respectively. Notably, POP1 exhibited the slowest decay velocity, while POP3 displayed the fastest decay velocity (Supplementary Table S1).

### 3.6. Discovery of Marker–Trait Associations and Favorable Alleles for the Eight Traits in a Natural Population

In the current study, we adopted a precise selection criterion by excluding all accessions with a Q value less than 0.9, resulting in a subset of 468 accessions used for marker–trait association mapping. To perform this analysis, we employed the MLM (mixed linear model) approach for marker–trait associations, utilizing the Q + K model within TASSEL 3.0, with a significance threshold set at *p* < 0.05. This rigorous analysis successfully identified 106 marker loci that displayed significant associations with the eight traits under investigation, which were evaluated over two years (Supplementary Table S3). Among these markers, 59 were found to be consistently associated with the eight traits in both experimental years (2021 and 2022), as described in Supplementary Table S5. These markers have also been previously documented in published studies (accessible through http://www.gramene.org/markers/ on 15 October 2023), and their associations were further verified using (http://:blast.ncbi.nlm.nih.gov/Blast.cgi, accessed on 15 October 2023) and the China Rice Data Center database (http://www.ricedata.cn/gene/list/1499.htm, accessed on 15 October 2023).

Our investigation classified marker alleles with positive effects as favorable for SD, AT/S, and FirINL traits. Conversely, alleles with adverse effects were deemed elite for PH, SecINL, ThirINL, ForINL, and FifINL traits.

This study identified a total of 185 favorable alleles, including 46 novel loci (Supplementary Table S4).

### 3.7. SSR Association Loci and Favorable Alleles for Various Plant Traits

#### 3.7.1. Plant Height in the Natural Population

We identified nineteen SSR association loci distributed across chromosomes 1, 2, 3, 4, 5, 6, 8, and 10 that exhibited associations with PH in the natural population. These loci displayed phenotypic variance explained (PVE) values ranging from 1.98% to 7.50%. Remarkably, RM5753, located on chromosome 6 at 124.4 cM, exhibited the highest PVE, accounting for 7.50% of the variation in PH in 2021 and 6.93% in 2022 (Supplementary Figure S5 and Supplementary Table S3).

We also identified a total of 24 favorable alleles associated with PH. Among these, the allele RM6811-160 bp exerted the most substantial phenotypic effect, resulting in a reduction of 22.79 cm in plant height when substituted with RM6811-000 bp. Notably, the accession younang429 was the typical carrier of this favorable allele.

These identical alleles associated with plant height were also linked with ThirINL, and the typical carrier accession for ThirINL was Cbao (Supplementary Table S4).

#### 3.7.2. Stem Diameter in the Natural Population

An extensive study on SD in the natural population identified thirteen significant marker loci (Supplementary Table S3, Supplementary Figure S5) across chromosomes 1, 2, 3, 5, 8, and 12, each demonstrating notable associations with this trait. These markers exhibited wide-range phenotypic variance explained (PVE) values, spanning from 2.23% to 7.97%.

One particular marker, RM5479, situated on chromosome 12 at 94.4 cM, is the most significant contributor to SD variation [44], boasting the highest PVE of 7.97% in 2021 and 7.87% in 2022. This marker played a pivotal role in elucidating the genetic basis of SD (Supplementary Figure S5 and Supplementary Table S4).

Furthermore, our analysis unveiled 22 favorable alleles associated with SD across the entire population. Among these alleles, RM161-145 bp displayed a substantial phenotypic effect by increasing stem diameter by a remarkable 0.159 mm. This advantageous allele’s typical carrier accession was huaidao11 (Supplementary Table S4).

#### 3.7.3. Stem Anti-Thrust in the Natural Population

Investigation into the genetic basis of anti-thrust of the stem (AT/S) revealed the presence of fifteen marker loci distributed across chromosomes 1, 2, 3, 5, 6, 7, 8, 10, and 11, all of which exhibited significant associations with this important agronomic trait. These markers displayed broad phenotypic variance explained (PVE) values, ranging from 3.29% to 8.46%, underscoring the genetic diversity underpinning AT/S regulation.

One marker, RM162 on chromosome 6 at 114.9 cM, emerged as a prominent driver of AT/S variation, demonstrating the highest PVE among all markers. Specifically, it accounted for 6.82% of the AT/S variation in 2021 and 6.61% in 2022 (Supplementary Figure S5 and Supplementary Table S3).

Furthermore, our analysis unveiled a remarkable overlap in genetic associations, as markers RM15356, RM168, and RM505 were not only linked to AT/S but also to other vital agronomic PH-related traits, including (PH), (ThirINL), (ForINL), and (FifINL). (Supplementary Figure S5 and Supplementary Table S3).

We identified 25 favorable alleles associated with AT/S across the entire population. Among these, the allele RM162-305 bp conferred the most substantial effect by increasing AT/S by 3.02 kPa. Tiejingqing was identified as the typical carrier accession for this vital allele.

Moreover, the favorable allele RM160-180 bp was found to be associated not only with AT/S but also with ThirINL, and its typical carrier accessions were yuedao37 and Cbao, respectively. Additionally, RM282-125 bp exhibited associations with AT/S and FifINL, with maijieqing and tiejingqing identified as their specific carrier accessions, respectively (Supplementary Table S4).

#### 3.7.4. First Internode Length Trait (FirINL) in the Natural Population

The first internode length trait revealed genetic associations with ten marker loci positioned across chromosomes 1, 3, 4, 6, 8, 10, and 12 (Supplementary Table S3, Supplementary Figure S5). These markers exhibited diverse phenotypic variance explained (PVE) values, ranging from 2.21% to 9.24%.

One specific marker, RM48, situated on chromosome 1, significantly influences FirINL variation. It displayed a substantial PVE of 7.21% in 2021 and an even higher 9.24% in 2022, as illustrated in Supplementary Figure S5 and documented in Supplementary Table S3.

Furthermore, our analysis showed 18 favorable alleles associated with FirINL across the population. Among these alleles, RM48-195 bp exhibited the most significant phenotypic effect by increasing FirINL by a notable length of 7.87 cm. The typical carrier accession for this allele was identified as kendao.

Notably, the same allele, RM48, was also associated with stem diameter (SD), with the typical carrier accession being heitouhong (Supplementary Table S4).

#### 3.7.5. Second Internode Length Trait (SecINL) in the Natural Population

The SecINL trait displayed associations with ten marker loci distributed across chromosomes 1, 2, 4, 6, 8, 10, and 11. These markers exhibited a range of phenotypic variance explained (PVE) values, spanning from 2.27% to 8.55%, providing key insights into the genetic regulation of SecINL.

Among these markers, RM6215, located on chromosome 8 at 66.8 cM, was identified as the key contributor to SecINL variation. This marker demonstrated the highest PVE, precisely 7.35% in 2021 and an even more substantial 8.55% in 2022 (Supplementary Table S3, Supplementary Figure S5).

Further analysis revealed the presence of 23 favorable alleles associated with SecINL across the entire population. Notably, the allele RM1019-120 bp displayed the most significant phenotypic effect, resulting in a reduction of −1.57 cm in SecINL. The typical carrier accession for this advantageous allele was identified as erheidao, underscoring its importance in SecINL regulation (Supplementary Table S4).

#### 3.7.6. Third Internode Length Trait (ThirINL) in the Natural Population

The investigation of ThirINL identified associations with eight marker loci and six new QTLs positioned across chromosomes 1, 2, 3, 6, 10, and 11. These markers exhibited a range of phenotypic variance explained (PVE) values, spanning from 2.27% to 6.18%, shedding light on the genetic factors influencing ThirINL.

Among these markers, RM168, located on chromosome 3 at 122.8 cM, was depicted as a significant contributor to ThirINL variation. It demonstrated the highest PVE, precisely 7.97% in 2021 and 7.87% in 2022. This marker was also associated with the fourth internode length (ForINL) (Supplementary Table S3, Supplementary Figure S5).

Furthermore, our analysis revealed the presence of 20 favorable alleles associated with ThirINL across the entire population. Remarkably, the allele RM8095-190 bp displayed the most substantial phenotypic effect, resulting in a reduction of −2.26 cm in ThirINL. The typical carrier accession for this allele was identified as longjing26, underscoring its importance in ThirINL regulation (Supplementary Table S4).

#### 3.7.7. Fourth Internode Length (ForINL) in the Natural Population

Our comprehensive ForINL study identified associations with eighteen marker loci distributed across chromosomes 1, 2, 3, 4, 5, 6, 8, 9, 10, 11, and 12. These markers exhibited a range of phenotypic variance explained (PVE) values, spanning from 1.85% to 7.55%, revealing the intricate genetic influences shaping ForINL.

Among these markers, RM206, located on chromosome 11 at 102.9 cM, significantly contributed to ForINL variation. It exhibited the highest PVE of 7.55% in 2021 and 7.00% in 2022 (Supplementary Figure S4 and Supplementary Table S3). Moreover, our analysis unveiled 25 favorable alleles associated with ForINL across the entire population. Notably, the allele RM212-135 bp showcased the most substantial phenotypic effect, resulting in a reduction of −3.63 cm in ForINL. The typical carrier accession for this allele was identified as yue6. Interestingly, these identical alleles were also found to be associated with other traits, such as plant height (PH) and fifth internode length (FifINL), with the typical carrier accession being wuxidao (Supplementary Table S4).

#### 3.7.8. Fifth Internode Length Trait (FifINL) in the Natural Population

A thorough examination of the FifINL trait identified associations with thirteen marker loci dispersed across chromosomes 1, 2, 3, 4, 6, 7, and 8. These markers exhibited diverse phenotypic variance explained (PVE) values, spanning from 2.59% to 7.75%.

One specific marker, RM6314, located on chromosome 4, significantly contributes to the FifINL variation. It displayed the highest PVE, precisely 7.75% in 2021 and 6.87% in 2022. This same marker was also associated with FifINL and FirINL (Supplementary Table S3 and Supplementary Figure S5).

Furthermore, our analysis unveiled the presence of 20 favorable alleles associated with FifINL across the entire population. The allele RM212-80 bp exhibited the most substantial phenotypic effect, resulting in a reduction of −4.26 cm in FifINL. The typical carrier accession for this advantageous allele was identified as maijieqing (Supplementary Table S4).

### 3.8. New QTLs Detected for the 8 Traits

**Figure 5:**
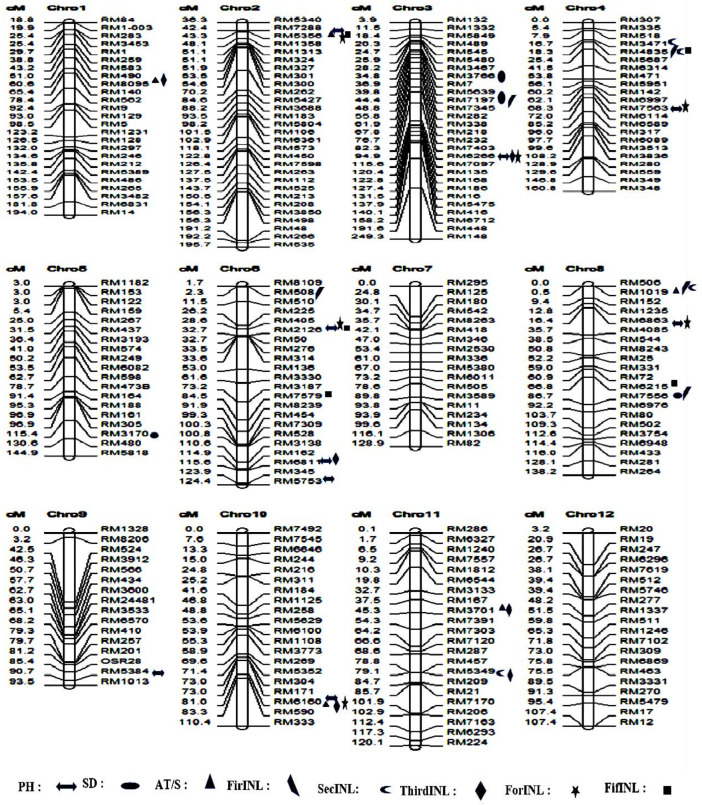
Chromosome positions of the newly identified QTLs for eight traits related to lodging resistance; the markers associated with the traits have symbols on the right side.

In this study, a total of 42 novel quantitative trait loci (QTLs) were discerned. Specifically, seven QTLs were identified for PH, four for SD, five for AT/S, five for FirINL, six for SecINL, five for ThirINL, six for ForINL, and four for FifINL (Supplementary Table S8 and Figure 5). Notably, we uncovered pleiotropic QTLs that exhibit associations between traits, namely *qPH2.1*, *qAT/S2.1*, *qForINL2.1*, and *qFifINL2.1* located on chromosome 2. Additionally, *qAT/S8*, *qFirINL8.1*, *qSedINl8.1* on chromosome 8, *qSedINl8.2*, *qFifINL8* on chromosome 8, qFirINL4.1, qSedIN4.2 on chromosome 4, and *qPH6.1*, *qThirINL6* on chromosome 6 were identified as QTLs demonstrating complex interactions between genomic regions and traits.

### 3.9. Parental Combinations Predicted for Lodging-Resistant Improvement

Considering the potential for combining multiple favorable alleles within a single plant and the expected phenotypic improvements, we have identified eight distinct cross-combinations for enhancing each trait, as outlined in Supplementary Table S6. These proposed combinations leverage the advantageous alleles the parental lines carry, as listed in Supplementary Table S7. Among the promising parental varieties, ‘Xudao’ × ‘Baobao’, ‘Buxienuo’ × ‘Aibaidao’, ‘Wuxidao’ × ‘Zaoyedao’, ‘Hongbaodao’ × ‘kendao2’, and ‘Maijieqing’ × ‘Cai1’ were consistently significant. These combinations repeatedly possessed favorable alleles, showcasing their potential for significantly improving target traits.

## 4. Discussion

The analysis of variance unveiled highly significant differences among cultivars for each trait at a statistical significance level of *p* < 0.01. This robust statistical outcome underscores substantial genetic variation within the population under scrutiny. The genetic relatedness analysis revealed that over 89% of the kinship coefficient values (K matrix) fell within the range of 0.35–0.50. The remaining 9.8% of values indicated various degrees of genetic relatedness between pairwise accessions. This suggests the presence of significant relatedness among the accessions employed in the study. Notably, the AMOVA and *Fst* analyses were consistent with the results obtained from phylogenetic tree-based and STRUCTURE analyses, collectively highlighting the presence of statistically moderate genetic diversity and a highly diverse population structure in this study. The mean D′ value, a measure of LD, was observed to be highest in POP1, at 0.64, while the lowest value of 0.54 was recorded in POP3. These findings suggest that some accessions within specific subpopulations may have undergone substantial artificial selection pressures. POP1 exhibited the slowest decay velocity, while POP3 displayed the fastest decay velocity, indicating that accessions collected from Vietnam had undergone extensive recombination.

Notably, the average CV of FifINL across the two environments was higher than that of the other eight traits, indicating a significant variation in this trait among the studied population.

In this study, heritability in the broad sense for AT/S averaged 80.31% over two years, which is evidence of a remarkably high heritability. These findings align with previous studies [10,45]. Consequently, predictable enhancements in AT/S can be achieved through marker-assisted selection (MAS).

Of the fifteen SSR-associated markers identified for AT/S, marker RM5356 on chromosome 2 and RM505 on chromosome 7 were simultaneously associated with AT/S, PH ForINL, and FifINL. Similarly, RM162 on chromosome 6 and RM184 on chromosome 10 were concurrently associated with AT/S and SecINL. Additionally, the allele RM282-125 bp was found to overlap with AT/S and FifINL at their respective marker loci. Furthermore, additional loci influencing AT/S were mapped to the same genomic regions as internode length. RM5356, named qAT/S2.1, was found as a unique novel QTL identified and associated with AT/S, PH ForINL, and FifINL in this study, describing the lodging property through the AT/S trait. This observation suggests that the genetic loci governing AT/S and internode length share pleiotropic alleles, underscoring a significant relationship between stem anti-thrust and stem internode length [46,47,48].

Among the fifteen marker loci associated with AT/S, RM162 on chromosome 6 exhibited the highest significance, accounting for 6.82% of the variation. This marker, specifically the RM162-305 bp allele, was linked to a 3.02 kPa increase in AT/S, with the typical carrier material being tiejinqing. These discoveries deepen our understanding of the genetic mechanisms underlying AT/S and related agronomic traits. Furthermore, they highlight the potential utility of these marker alleles in crop-breeding efforts aimed at enhancing AT/S.

We identified a total of 106 QTLs associated with lodging traits. To compare these findings with previous research, we utilized whole-genome marker resources available on the Rice Gramene website (http://www.gramene.org/). We examined the chromosomal regions of SSR markers linked to lodging resistance and cross-referenced them with prior studies. Sixty-one of the identified QTLs had been previously detected and reported in earlier research. These included nine QTLs for AT/S [21,49,50], ten for PH [21,49,51], nine for SD [52,53,54,55], five for FirINL [15,56,57], five for SecINL [58], two for ThirINL [19,59], twelve for ForINL [60,61], and eight for FifINL [62,63,64].

Apart from the 61 SSR loci, we also identified 42 novel QTLs in our study. These novel QTLs encompassed various lodging component traits, including six for AT/S, nine for PH, four for SD, five for FirINL, five for SecINL, six for ThirINL, six for ForINL, and five for FifINL.

The marker RM5753 on chromosome 6 was the most significant, explaining 7.50% of the PH variation. Additionally, 24 favorable alleles for PH were identified, with RM6811-160 bp having the most substantial effect, reducing plant height by 22.79 cm. On the other hand, thirteen significant markers were identified for SD, with RM5479 on chromosome 12 being the most influential, explaining 7.97% of the variation. Twenty-two favorable alleles were identified, with RM161-145 bp increasing stem diameter by 0.159 mm. These findings provide insights into the genetic basis of SD and plant height and their potential for crop improvement.

Fifteen markers on various chromosomes were associated with AT/S. RM162 on chromosome 6 had the maximum PVE for AT/S (6.82% in 2021 and 6.61% in 2022) and was also associated with SecINL. A total of 25 favorable alleles for AT/S were identified, with RM5340 bp having the largest effect (4.11 kPa) and being typically carried by hongyin1012.

Nine markers on different chromosomes were associated with FirINL. RM48 on chromosome 1 had the highest PVE (7.21% in 2021 and 9.24% in 2022). A total of 18 favorable alleles for FirINL were detected, with RM48-195 bp showing the most significant phenotypic effect (7.87 cm) and being typically carried by kendao. Ten marker loci were associated with SecINL, with RM6215 on chromosome 8 being the most significant, explaining 8.55% of the variation. Twenty-three favorable alleles were identified, with RM1019-120 bp reducing SecINL by −1.57 cm. These findings provide insights into the genetic regulation of SecINL.

Eight marker loci were associated with ThirINL, with RM168 on chromosome 3 being the most prominent, explaining 7.97% of the variation. Twenty favorable alleles were discovered, with RM8095-190 bp reducing ThirINL by −2.26 cm. Genetic associations were also observed between ThirINL and other traits, such as ForINL.

Eighteen marker loci were associated with ForINL, with RM206 on chromosome 11 being the most influential, explaining 7.55% of the variation. Twenty-five favorable alleles were identified, with RM212-135 bp reducing ForINL by −3.63 cm. Genetic links between ForINL and other traits, including PH and FifINL, were also discovered.

Thirteen marker loci were associated with FifINL, with RM6314 on chromosome 4 being the most significant, explaining 7.75% of the variation. Twenty favorable alleles were found, with RM212-80 bp reducing FifINL by −4.26 cm. Genetic associations between FifINL and FirINL were also observed. These discoveries mine our understanding of the genetic mechanisms underlying FirINL and its potential interplay with other agronomic traits. Identifying favorable alleles, such as RM48-195 bp, not only enhances our knowledge but also opens up promising avenues for precision breeding programs to optimize FirINL and related traits for specific agricultural objectives.

This study identified eight distinct cross-combinations for enhancing each trait. This discovery suggests the potential for crossbreeding superior parents based on the number of alleles stacked within an individual plant, the expected phenotypic improvements in lodging resistance traits, and the anticipated benefits of favorable alleles. This strategic approach to crossbreeding considers the genetic advantages of different parental lines and offers a promising avenue for enhancing specific traits in crop-breeding programs. By capitalizing on the synergistic effects of multiple favorable alleles, we can work towards achieving improved crop performance and productivity.

These results provide useful insights into the genetic basis of various agronomic traits in the natural population, including identifying significant loci and favorable alleles. This knowledge can inform precision breeding programs to optimize these traits for specific agricultural goals, ultimately improving crop performance and yield.

## 5. Conclusions

The natural population in this study exhibited a substantial genetic diversity, with highly significant differences observed among the 518 accessions concerning stem lodging and its constituent traits. Our study identified 42 stable, previously unreported genetic loci associated with eight different traits. We detected qAT/S-2, which is linked with RM5356 and was also found to be associated with PH, ForINL, and FifINL. This discovery is particularly significant as qAT/S-2, influencing AT/S (stem lodging) in tandem with PH, ForINL, and FifINL, represents a unique QTL, showcasing rice’s resistance to bending or pushing forces.

Furthermore, our study unveiled several other novel QTLs, including two for SD and seven for FirINL, SecINL, and THirINL. These findings provide key research information into the potential for manipulating plant height architecture by combining favorable alleles distributed across various loci in rice. Additionally, they offer fresh perspectives on targets for enhancing lodging resistance in rice.

## Figures and Tables

**Table 1:** Descriptive statistics of PH and its component traits in natural population across 2 years.

Traits	Year	Mean ± SD	Min	Max	Skewness	Kurtosis	CV (%)	h^2^
PH (cm)	2021	111.23 ± 22.93	62.22	175.5	0.22	−0.89	20.62	0.99
	2022	112.20 ± 23.13	62.35	175	0.25	−0.89	21.79	0.99
SD (cm)	2021	7.4 1 ± 1.28	3.69	13.1	0.44	0.84	17.29	0.80
	2022	7.40 ± 1.29	3.69	12.9	0.41	0.82	15.38	0.80
AT/S (Kpa)	2021	9.06 ± 2.47	3.42	17.79	0.57	0.04	28.34	0.80
	2022	9.05 ± 2.48	3.42	17.79	0.56	0.03	27.44	0.80
FirINL (cm)	2021	34.86 ± 7.93	12.43	95.67	1.59	8.87	22.75	0.65
	2022	34.85 ± 7.93	12.4	95.66	1.59	8.87	20.95	0.64
SedINL (cm)	2021	23.46 ± 5.24	10.18	40.38	0.47	0.06	21.35	0.92
	2022	23.58 ± 5.22	10.32	38.06	0.43	−0.4	22.16	0.97
ThirINL (cm)	2021	19.31 ± 5.45	6.82	32.93	0.29	−0.87	28.35	0.94
	2022	19 ± 5.48	6.5	32.61	0.27	−0.87	28.85	0.94
ForINL (cm)	2021	14.1 ± 6.05	1.96	31.44	0.42	−0.75	42.91	0.93
	2022	14.49 ± 6.05	2.43	31.73	0.41	−0.77	41.73	0.92
FifINL (cm)	2021	8.42 ± 5.42	0.94	28.88	0.59	−0.66	45.35	0.92
	2022	9.13 ± 5.44	0.71	29.58	0.57	−0.67	46.63	0.92

PH, plant height; SD, stem diameter; AT/S, ant-thrust per stem; FirINL, first internode length from the top; SedINL, second internode length from the top; ThirINL, third internode length from the top; ForINL, fourth internode length from the top; FifINL, fifth internode length from the top, SD, standard deviation; CV, coefficient of variation; h^2^, heritability.

**Table 2:** Analysis of molecular variance (AMOVA) for the three subpopulations of rice varieties.

Source	df	SS	MS	Est. Var.	PMV%	*p*-Value
Among Pops	2	13,681.113	6840.557	19.919	19%	*p* < 0.01
Within Pops	466	87,608.867	85.140	85.140	81%	*p* < 0.01
Total	468	101,289.981		105.058	100%	

Pops, populations; df, degrees of freedom; SS, sum of squares; MS, mean square; Est. Var, estimate of the molecular variance; PMV, percentage of the variance.

**Table 3:** Pairwise population differentiation according to groups of populations as measured by *Fst* values using Arlequin software ver. 3.5.

Subpopulation	Pop1	Pop2	Pop3
Pop1		0.52	0.69
Pop2	0.56		0.58
Pop3	0.48	0.44	

Nei’s genetic distances appear above the diagonal, and pairwise Fst values appear below the diagonal. All *Fst* values are significant (*p* < 0.01).

**Table 4:** Comparison of D’ values for pairwise SSR in each subpopulation.

Cluster	No. of LD ^a^	Ratio ^b^	Frequency of D′ ^c^ Value (*p* < 0.05)					Means of D′
	Locus Pairs	(%)	0–0.2	0.2–0.4	0.4–0.6	0.6–0.8	0.8–1.0	
POP1	1240	2.7	160	250	271	370	302	0.64
POP2	725	4.7	96	266	265	145	193	0.61
POP3	1437	2.4	49	227	361	335	190	0.53

^a^ LD means linkage disequilibrium. ^b^ Ratio among the number of significant LD locus pairs and total number of LD locus pairs. ^c^ D’ denotes standardized disequilibrium.

## Data Availability

The relevant data sources related to this manuscript are available within this paper and in the supplementary material.

## Supplementary Materials

The following supporting information can be downloaded at: https://www.mdpi.com/article/10.3390/genes15010105/s1, Figure S1: Images showing measurements of stem anti-thrust, stem diameters Plant height and 1 to 5 internode lengths with their materials on the field; Figure S2: 518 rice variety belonging to Three subpopulations, calculated by STRUCTURE software. Each vertical bar represents an accession and within each vertical bar, the colored subsections represent membership coefficient (Q) of the accession to different clusters. Identified subpopulations are Sub-pop 1 (red color), Sub-pop 2 (green color) and Subpop 3 (navy blue color). The numbers in the X-axis stand for variety code corresponding to Table S1; Figure S3: Neighbor joining tree for the 518 accessions constructed using Nei’s (1983) genetic distance; Figure S4: Relationship between *D′* value and genetic distance of syntenic marker pairs in subpopulations POP1, POP2 and POP3 represent subpopulation 1 to subpopulation 3, respectively; Supplementary Figure S5: Genome-wide association showing significant loci associated with PH and its component traits in 2021 and 2022; Table S1: Rice materials and their membership probabilities corresponding to each subpopulation; Table S2: Summary statistics for the 262 SSR markers used in this study; Table S3: Marker-trait association with *p*-value less than 0.05, proportion of phenotypic variance explained (PVE), marker position on chromosome derived from 262 markers and 518 Rice Accessions; Table S4: phenotypic effect of top two positive elite alleles significantly associated with the 8 traits and their carrier varieties; Table S5: The list for QTLs identified from this study and shared in previous studies [65,66]; Table S6: Favorable alleles carried by the superior parents for the 8 traits and corresponding phenotypic effect; Table S7: Parental combinations and numbers of elite alleles after combinations predicted for the 8 traits; Table S8: Information of QTLs detected for the eight traits of this study.
