# Supplementary material for: Genome-Wide Association Analysis Unravels New Quantitative Trait Loci (QTLs) for Eight Lodging Resistance Constituent Traits in Rice (Oryza sativa L.)
Source: Genes (Basel). 2024 Jan 16;15(1):105. doi: 10.3390/genes15010105 (PMC10815206; doi:10.3390/genes15010105)
Supplement: Supplementary file 1 [file genes-15-00105-s001.zip › Supplementary Fig S1.pdf]

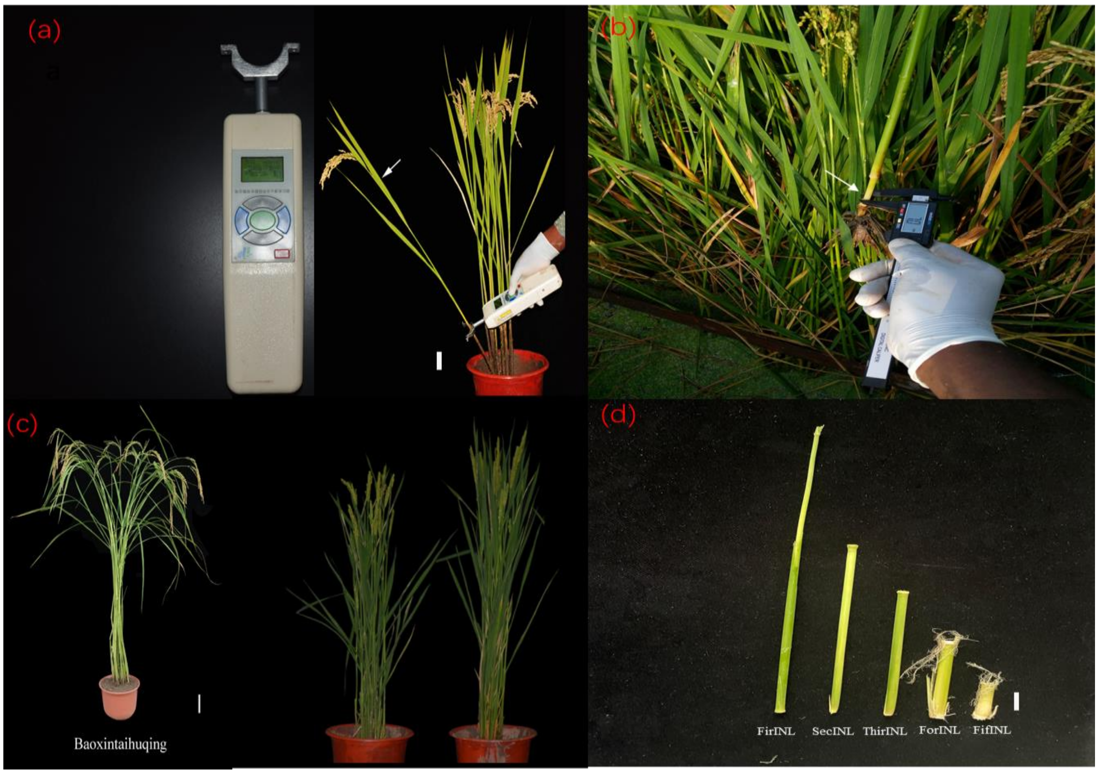

Figure S1 Images showing measurements of stem anti-thrust, stem diameters Plant height and 1 to 5 internode lengths with their materials on the field
