# Supplementary material for: Genome-Wide Association Analysis Unravels New Quantitative Trait Loci (QTLs) for Eight Lodging Resistance Constituent Traits in Rice (Oryza sativa L.)
Source: Genes (Basel). 2024 Jan 16;15(1):105. doi: 10.3390/genes15010105 (PMC10815206; doi:10.3390/genes15010105)
Supplement: Supplementary file 1 [file genes-15-00105-s001.zip › Supplementary Fig S2.pdf]

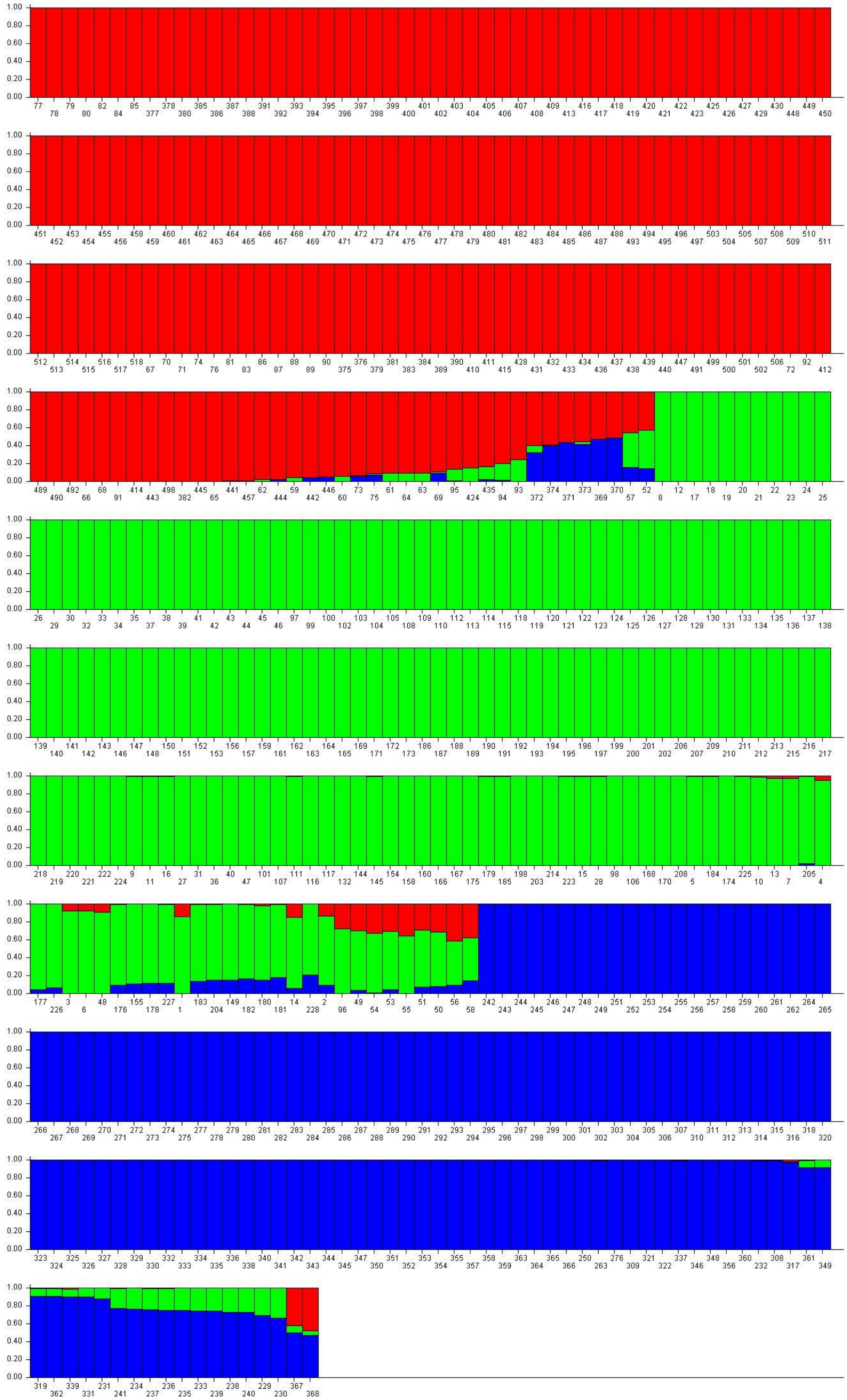
1.00
0.80
0.60
0.40
0.20
0.00
77 78 79 80 82 84 85 377 378 380 385 386 387 388 391 392 393 394 395 396 397 398 399 400 401 402 403 404 405 406 407 408 409 413 416 417 418 419 420 421 422 423 425 426 427 429 430 448 449 450
451 452 453 454 455 456 458 459 460 461 462 463 464 465 466 467 468 469 470 471 472 473 474 475 476 477 478 479 480 481 482 483 484 485 486 487 488 493 494 495 496 497 503 504 505 507 508 509 510 511
512 513 514 515 516 517 518 67 70 71 74 76 81 83 86 87 88 89 90 375 376 379 381 383 384 389 390 410 411 415 428 431 432 433 434 436 437 438 439 440 447 491 499 500 501 502 506 72 92 412
489 490 492 66 68 91 414 443 498 382 445 65 441 457 62 444 59 442 446 60 73 75 61 64 63 69 95 424 435 94 93 372 374 371 373 369 370 57 52 8 12 17 18 19 20 21 22 23 24 25
26 29 30 32 33 34 35 37 38 39 41 42 43 44 45 46 97 99 100 102 103 104 105 108 109 110 112 113 114 115 118 119 120 121 122 123 124 125 126 127 128 129 130 131 133 134 135 136 137 138
139 140 141 142 143 146 147 148 150 151 152 153 156 157 159 161 162 163 164 165 169 171 172 173 186 187 188 189 190 191 192 193 194 195 196 197 199 200 201 202 206 207 209 210 211 212 213 215 216 217
218 219 220 221 222 224 9 11 16 27 31 36 40 47 101 107 111 116 117 132 144 145 154 158 160 166 167 175 179 185 198 203 214 223 15 28 98 106 168 170 208 5 184 174 225 10 13 7 205 4
177 226 3 6 48 176 155 178 227 1 183 204 149 182 180 181 14 228 2 96 49 54 53 55 51 50 56 58 242 243 244 245 246 247 248 249 251 252 253 254 255 256 257 258 259 260 261 262 264 265
266 267 268 269 270 271 272 273 274 275 277 278 279 280 281 282 283 284 285 286 287 288 289 290 291 292 293 294 295 296 297 298 299 300 301 302 303 304 305 306 307 310 311 312 313 314 315 316 318 320
323 324 325 326 327 328 329 330 332 333 334 335 336 338 340 341 342 343 344 345 347 350 351 352 353 354 355 357 358 359 363 364 365 366 250 263 276 309 321 322 337 346 348 356 360 232 308 317 361 349
319 362 339 331 231 241 234 237 236 235 233 239 238 240 229 230 367 368

Supplementary Figure S2. 518 rice variety belonging to Three subpopulations, calculated by STRUCTURE software. Each vertical bar represents an accession and within each vertical bar, the colored subsections represent membership coefficient (Q) of the accession to different clusters. Identified subpopulations are Sub-pop 1 (red color), Sub-pop 2 (green color) and Subpop 3 (navy blue color). The numbers in the X-axis stand for variety code corresponding to Table S1.
