# Supplementary material for: Genome-Wide Association Analysis Unravels New Quantitative Trait Loci (QTLs) for Eight Lodging Resistance Constituent Traits in Rice (Oryza sativa L.)
Source: Genes (Basel). 2024 Jan 16;15(1):105. doi: 10.3390/genes15010105 (PMC10815206; doi:10.3390/genes15010105)
Supplement: Supplementary file 1 [file genes-15-00105-s001.zip › Supplementary Fig S5.pdf]

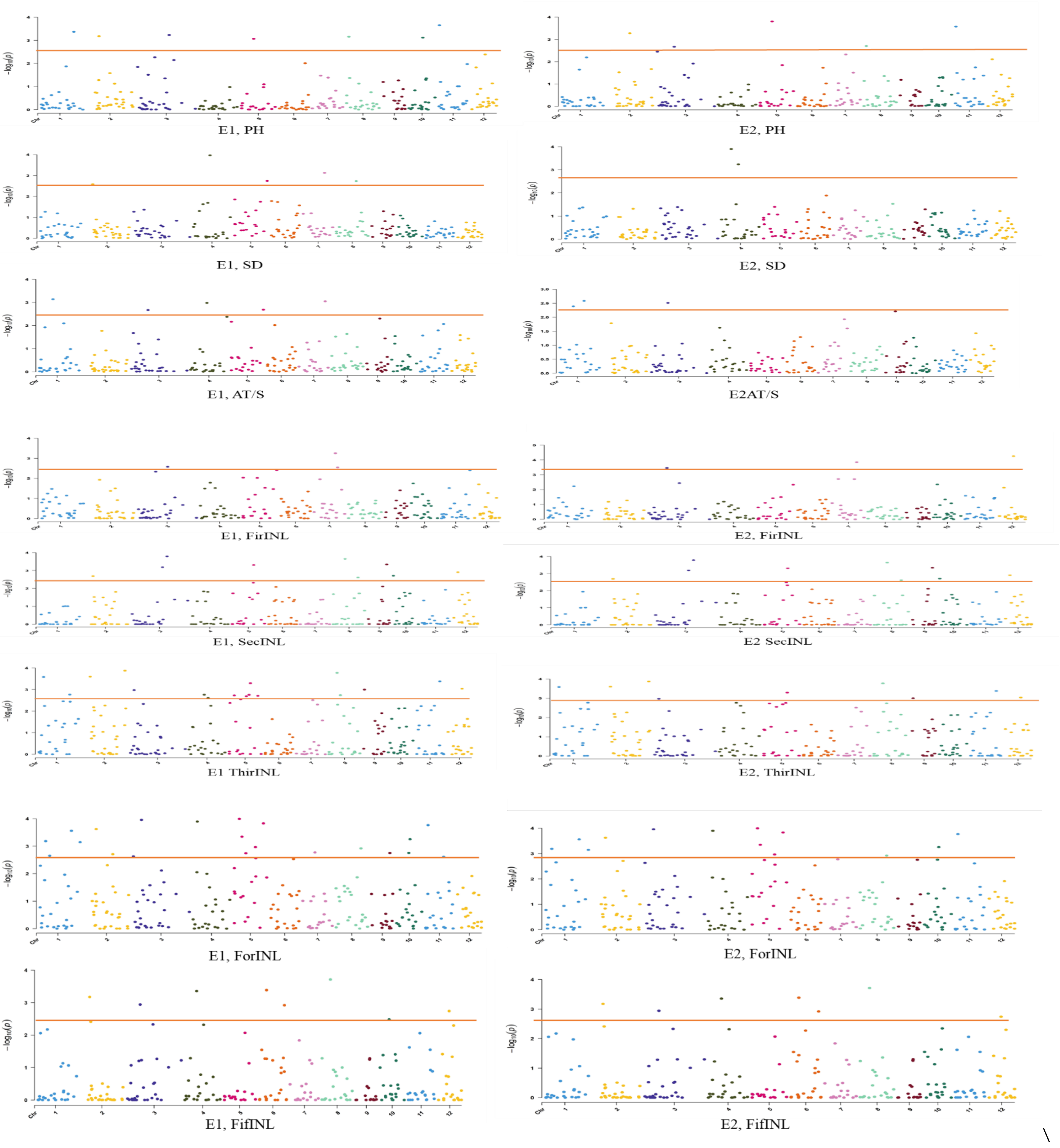

Supplementary Figure S5 Genome-wide association showing significant loci associated with PH and its component traits in 2021 and 2022
